# Acetabular cup orientation and postoperative leg length discrepancy in patients undergoing elective total hip arthroplasty via a direct anterior and anterolateral approaches

**DOI:** 10.1186/s12891-018-2097-4

**Published:** 2018-06-08

**Authors:** Ronen Debi, Evyatar Slamowicz, Ornit Cohen, Avi Elbaz, Omri Lubovsky, Dror Lakstein, Zachary Tan, Ehud Atoun

**Affiliations:** 1 Department of Orthopedic Surgery, Barzilai Medical Center, 2 Hahistadrut Street, 78278 Ashkelon, Israel; 2 AposTherapy Research Group, Herzelyia, Israel; 3 Department of Orthopaedic Surgery, Wolfson Medical Center , Holon, Israel; 4 Affiliated to the Ben-Gurion University of the Negev, Beer sheva, Israel

**Keywords:** THA, Approach, Anterolateral, Direct anterior, Anteversion, Abduction, Angle, Leg length discrepancy

## Abstract

**Background:**

Total hip arthroplasty (THA) is considered a successful surgical procedure. It can be performed by several surgical approaches. Although the posterior and anterolateral approaches are the most common, there has been increased interest in the direct anterior approach. The goal of the present study is to compare postoperative leg length discrepancy and acetabular cup orientation among patients who underwent total hip arthroplasty through a direct anterior (DAA) and anterolateral (ALA) approaches.

**Methods:**

The study included 172 patients undergoing an elective THA by a single surgeon at our institution within the study period. Ninety-eight arthroplasties were performed through the ALA and 74 arthroplasties through the DAA. Preoperative planning was performed for all patients. Assessment of the two groups included the following postoperative parameters: abduction angle, cup anteversion angle and leg length discrepancy (LLD). Additional analysis was done to evaluate component positioning by comparing deviation from the Lewinnek zone of safety in both approaches.

**Results:**

For the DAA the absolute LLD was 11 mm, ranging from -6 mm to 5 mm. For the ALA, the absolute LLD was 36 mm, ranging from -22 mm to 14 mm. None of the DAA patients had an absolute LLD greater than 6 mm. Comparatively, 7.4% of the ALA group exceeded 6 mm of LLD in addition to 2.1% with LLD greater than 10 mm. 15% of the ALA group resided out of the Lewinnek abduction zone compared to 3% of the DAA group (*P* = 0.016). 17% of the ALA group were out of the Lewinnek anteversion zone as opposed to 8% of the DAA group (*P* = 0.094).

**Conclusion:**

Our study demonstrates good component positioning outcomes and LLD values in patients following THA through the DAA compared to the ALA.

## Background

Total hip arthroplasty (THA) is considered a successful surgical procedure for the treatment of end-stage hip osteoarthritis. It offers pain relief and significant improvement in patient function and quality of life [1]. The number of THA performed worldwide is expected to increase in the following decades due to increasing life expectancy [2].

THA is performed by several surgical approaches. Currently, the posterior and anterolateral approaches are the most commonly used worldwide. Data extracted from the global orthopedic registry in 2010 indicate that 55 and 33% of THA are performed using a posterior approach and anterolateral approach (ALA) respectively [3]. Since 2013, there has been a renewed interest in the direct anterior approach (DAA) - 10% of orthopedic arthroplasty surgeons consider it their preferred approach [4]. Reduced blood loss, rapid functional recovery, low dislocation rates and shorter hospital stays have been attributed to the muscle-sparing properties of the anterior approach [5].

Two recent systematic reviews and network meta-analysis aimed to evaluate the effectiveness of surgical approach for THA (posterior, posterior-2, anterolateral, direct lateral, and anterior). Outcome measures were the length of the incision, blood loss, operating time, length of stay, complications, gait analysis and post-operative clinical symptoms [6, 7]. However, both reviews did not compare the acetabular cup positioning and postoperative leg length discrepancy. The latter were found to correlate with pain, excessive wear and instability [8]. Some evidence suggests that both the anterolateral and anterior approaches were found to safely, reliably and accurately produce an optimal component positioning, as well as clinical leg length discrepancy [9–11].

The goal of our study was to further examine the effect of the surgical approach on postoperative leg length discrepancy and acetabular cup positioning in patients undergoing a total hip arthroplasty through either a DAA or ALA. We hypothesized that there would be no significant difference between these two groups.

## Methods

Approval from Barzilay Medical Center Ethics Committee (IRB 0116–15-BRZ) was obtained.

A retrospective chart review was performed of patients undergoing an elective THR between January 2011 and December 2015 in our institute. The surgical approach was selected according to the availability of the dedicated DAA surgical table. Patients with extraarticular deformity, prior hip surgery, and contralateral THR were excluded from the study. One hundred fifty-eight patients were ultimately included, with 94 and 64 in the ALA and the DAA, respectively. All surgeries were performed by a single, fellowship trained orthopedic surgeon.

### Surgical technique

#### Direct anterior approach

Surgery is performed in the supine position. Both patient’s feet are secured into special boots connected to the DAA table. The skin incision starts approximately 3 cm lateral and 1 cm distal to the ipsilateral anterior superior iliac spine. Deep dissection is performed parallel to the fibers of the tensor fascia lata muscle. The fascia over the tensor muscle is incised and extended proximally and distally. The Smith-Peterson interval between the Tensor and the Sartorius muscles is used to access the joint. The ascending branch of the lateral femoral circumflex vessels are identified and cauterized. An L shaped hip capsulotomy is performed. The femoral neck is then osteotomized with the subsequent extrication of the femoral head. The neck cut is then verified in reference to the superior margins of the lesser trochanter. Intraoperative X-Ray imaging was utilized to ensure leg lengths and acetabular component positioning.

#### Anterolateral approach

Surgery is performed in the lateral decubitus position. A longitudinal skin incision is made over the lateral proximal thigh. The Tensor Fascia Lata is divided in line with the skin incision. Gluteus medius and Gluteus minimus are split in line with their fibers and a T shaped hip capsulotomy is performed. The soft tissue around the femoral neck is elevated and the hip is dislocated anteriorly. The lesser trochanter is then exposed. The femoral neck is osteotomized at the pre-planned level. Neck length is verified in reference to the superior margins of the lesser trochanter. Intraoperative XR or fluoroscopy was not used in the setting of the ALA approach.

#### Component implantation and closure

All patients received cementless Corail stems and Pinnacle cups (Depuy, Warsaw, Indiana). Intraoperatively, hip stability was assessed in several ways. Combined anteversion was assessed with the trial stem and adjusted as needed. Anterior and posterior stability was interrogated at extremes of range of motion and impingement was excluded. Stem offset was established during preoperative planning and verified using the palpable tension of the glutei and fascia lata after trial reduction. It is the surgical team’s uniform preference to accept 3 to 5 mm of “push-pull” joint laxity unless hip stability is grossly compromised. The joint capsule is sutured back and the glutei are meticulously repaired. There were no enforced hip precaution protocols.

#### Post operatively

All patients underwent pre and postoperative pelvic digital radiography via a standard protocol. The pelvic AP views were taken with the patient supine, the XR beam centered over the pubic symphysis with both hips internally rotated 10 to 15 degrees to offset physiologic anteversion. Routine pre-operative planning was performed using dedicated software (TraumaCad, Voyant health, Petach-Tikva, Israel) [12].

**Fig. 1:**
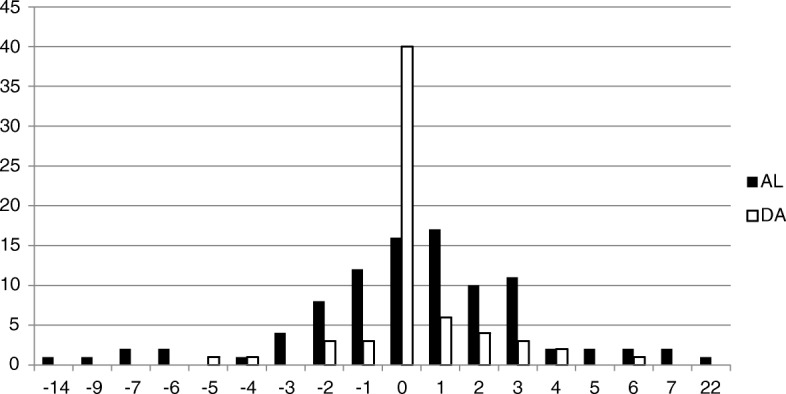
Leg length discrepancy (mm) in the DAA and ALA approaches

The software was used to measure LLD, cup anteversion and cup abduction for each patient. The in situ prosthetic femoral head with its documented diameter was used for radiographic calibration. The LLD was measured as the difference of the perpendicular vertical displacement from the tangential ischial line to a consistent point of reference on the lesser trochanter when comparing both hips. The software extrapolates anteversion by measuring the area of the elliptical projection of the cup - the greater the area, the greater the magnitude of the anteversion. Cup abduction is calculated as the angle between the horizontal inter-teardrop line and the cup obliquity (Fig. 1). Lewinnek’s standard safe-zone of abduction and anteversion angles range were considered (21). The range for anteversion angle is between 5 and 25 degrees, and for abduction angle is between 30 and 50 degrees.

The data distributions were examined using Kolmogorov-Smirnov tests for normality.

This study had 94 patients in the ALA group and 64 patients in the DAA group. In a previous study, the response within each subject group was normally distributed with a standard deviation of 6.5. If the true difference in the experimental and control means is 3.65, we will be able to reject the null hypothesis that the population means of the experimental and control groups are equal with a probability (power) of .932. The Type I error probability associated with this test of this null hypothesis is .05.

Statistical analysis was carried out with a one-way ANOVA test and the Enter method for correlation regression analysis (SPSS 21.0, SPSS Inc.) to examine the difference between digital radiographic measurements. *P*-values < 0.05 were considered significant.

## Results

There was no statistically significant difference between the ALA group and the DAA group with regards to gender, age, and body mass index (BMI). (Table 1).

**Table 1:** Population characteristics

	ALA	DAA	*p*-value
Mean age (SD)	64.8 (10.3)	64.5 (11)	0.875
Gender (F:M)	58:36	33:31	0.205
Mean BMI (SD)	29.2 (4.9)	28.7 (4.4)	0.501

*ALA* Anterolateral approach, *DAA* Direct anterior approach

**Fig. 2:**
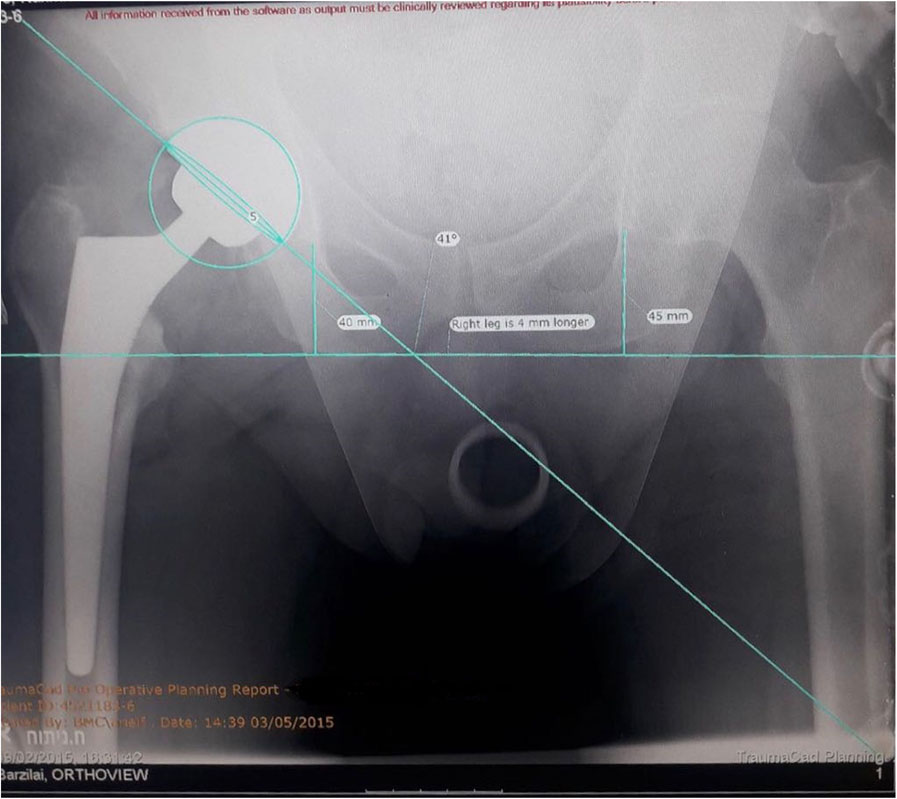
AP view of cup position assessment

The LLD measurements in the DAA group was distributed within a total range of 11 mm from *-6 mm to 5 mm* compared to a total range of 36 mm from -22 mm to 14 mm for the ALA group. None of the DAA patients had an absolute LLD greater than 6 mm. Comparatively, 7.4% of the ALA group exceeded 6 mm of LLD in addition to 2.1% with LLD greater than 10 mm (Fig. 2).

Absolute LLD was found to be significantly lower (*P* = 0.001) in the DAA group. The average absolute LLD value of the DAA approach was 0.9 mm whereas the average discrepancy in the ALA was 2.4 mm (Table 2).

**Table 2:** Acetabular component positioning and leg length discrepancy

	ALA (*n* = 94)	DAA (*n* = 64)	*p*-value
Mean anteversion Angle (°) (SD)	11.3 (6.6)	15 (5.5)	< 0.001
Mean Abduction Angle (°) (SD)	36.5 (5.9)	38.3 (6.1)	0. 069
Mean absolute LLD (mm) (SD)	2.4 (3.1)	0.9 (1.4)	0.001

*ALA* Anterolateral approach, *DAA* Direct anterior approach

In 96.9% of the cases in the DAA group, the cup anteversion angle was within the Lewinnek zone as compared to 85.1% in the ALA group (*P* = 0.016). In 92.2% of the cases in the DAA group, the cup abduction angle was within the Lewinnek zone as compared to 83% in the AL approach (*P* = 0.094) (Table 3). The cup anteversion angle was significantly higher in the DAA group. There was no significant difference in cup abduction angle between two groups (Table 2).

**Table 3:** Comparison of the Lewinnek target zone

		ALA (*n* = 94)	DAA (*n* = 64)	*p*-value
Abduction (%) (n)	in zone	85.1 (80)	96.9 (62)	0.016
	out of zone	14.9 (14)	3.1 (2)	
Anterversion (%) (n)	in zone	83 (78)	92.2 (59)	0. 094
	out of zone	17 (16)	7.8 (5)	

Abduction zone: 30°-50°, Anteversion zone: 5°-25°

*ALA* Anterolateral approach, *DAA* Direct anterior approach

## Discussion

The use of the direct anterior approach for THA has become increasingly popular in recent years [4]. Its muscle sparing principles are suggested to be associated with shorter hospital stays, higher rates of patients discharged home and better short-term postoperative outcomes. Overall, complication rates in the available literature do not appear to exceed those of the conventional approaches for THA [13]. A meta-analysis performed by Higgins et al. comparing the clinical and surgical outcomes in patients undergoing THA via the DAA and posterior approaches favored the DAA in the metrics of post-operative pain, functional outcomes, length of hospitalization, hip stability and postoperative narcotic consumption [14]. The PRISMA meta-analysis comparing direct anterior and lateral approaches similarly suggested that DAA may be associated with improved early postoperative functional rehabilitation, lower levels of perceived pain, and shorter hospitalization time [15]. A recent network meta-analysis by Putananon et al. evaluated all surgical approaches for THA [6]. They suggest that a failure of fixation, instability, and damage to soft tissues, associated with the trauma of the surgical procedure are some of the reasons for post-operative pain, which prevent patients to return to full function and activity. However, in their report as well as another meta-analysis, less attention is given to the acetabular cup positioning and postoperative leg length discrepancy, which in our opinion are important when evaluating the effectiveness of the surgical approach.

Patient dissatisfaction due to LLD is common. Complications associated with LLD include gait disorders, nerve injury, lower back pain, hip instability and occasionally necessitating surgical revision. The amount of discrepancy that is clinically acceptable following THA is controversial. Several studies showed that most patients tolerated LLD up to 10 mm while others reported that even a small discrepancy could produce dissatisfaction [16]. The varied opinions in the literature can be attributed to having significant LLD increase both *technical* post-operative complications and cause *subjective* patient dissatisfaction, both of which may occur at a different length thresh-holds [17–19]. The results of our study showed that the LLD absolute range in the DAA group was 11 mm, ranging from *-6 mm to 5 mm* compared to a total range of 36 mm, ranging from -22 mm to 14 mm for the ALA group. None of the DAA patients had an absolute LLD greater than 6 mm. Comparatively, 7.4% of the ALA group exceeded 6 mm of LLD in addition to 2.1% with LLD greater than 10 mm (Fig. 1).

The orientation of the acetabular component greatly influences hip joint stability after THA. Postoperative radiological measurements of abduction and anteversion angle are a common method for evaluating acetabular component orientation [20]. A second method for evaluating acetabular component orientation is to assess cup placement in a target zone. Lewinnek et al. [21] defined a “safe zone” that minimizes instability after THA comprising of both cup abduction and anteversion within 40° ± 10° and 15° ± 10°, respectively. Dislocation rates are demonstrably higher with components residing outside the defined “safe zone”. The Lewinnek concept of “safe zone” has to be a matter of recent controversy [22, 23], nevertheless, it remains an accessible method for confirming technical accuracy after THA [24]. In the current study, a higher percentage of the patients were within the defined “safe zone” in the DAA group compared to the ALA group (96.9% compared to 85.1% of cases, respectively, for cup anteversion angles and 92.2% compared to 83% of cases, for cup abduction angles). A possible explanation for the favorable accuracy of the DAA might be the intraoperative imaging that allows immediate feedback to the surgeon. The use of image intensification as verification of cup abduction and anteversion angles is technically easier in the supine position and allows components to be seated under sequential image guidance. This ensures acetabular component positioning and leg lengths [11]. In contrast, the ALA is performed in the lateral decubitus position, which complicates the acquisition of optimal and representative intraoperative imaging. The fact that only patients in the DAA had intraoperative x-ray may be a limitation to the current study, hence results and conclusions should be carefully considered. However, obtaining an intra-operative x-ray view of the component positioning in a lateral approach is more challenging and should be considered a limitation as well.

This study has several limitations. Firstly, although the DAA more frequently achieves better radiographic results than the ALA, both methods were found to be reasonably good in the setting of patient care. The differences, although statistically significant, are not enough to draw conclusions regarding clinical outcomes. Careful pre-operative templating and surgical experience may be the overriding factor regardless of approach [25]. Secondly, we acknowledge that missing information on intraoperative and postoperative complications, quality of life, and need for revision, is a limitation of the current study. However, Connolly and Kamath concluded that when surgeons have performed a modest number of procedures, the complication rates tend to markedly decrease in most studies to levels comparable to other approaches [13]. In the current study, all THAs were performed by a single, fellowship trained orthopedic surgeon, with > 100 cases experience in the DAA. Hence, it may be assumed that the frequency of complications rate did not differ between groups. Our purpose was to bring to the surgeon’s attention the importance of evaluating intraoperative component positions and LLD to improve surgery success. From our experience, these two parameters are easier to control and achieve good outcomes when using the DAA approach, however, we cannot determine that these cannot be achieved in other approaches.

## Conclusion

Our study demonstrates good component positioning outcomes and LLD values in patients following THA through the DAA compared to the ALA.

## Abbreviations

ALA: Anterolateral approach
BMI: body mass index
DAA: Direct anterior approach
THA: Total hip arthroplasty
